# Novel Regulation of Alpha-Toxin and the Phenol-Soluble Modulins by Peptidyl-Prolyl *cis/trans* Isomerase Enzymes in *Staphylococcus aureus*

**DOI:** 10.3390/toxins11060343

**Published:** 2019-06-16

**Authors:** Rebecca A. Keogh, Rachel L. Zapf, Emily Trzeciak, Gillian G. Null, Richard E. Wiemels, Ronan K. Carroll

**Affiliations:** 1 Department of Biological Sciences, Ohio University, Athens, OH 45701, USA; rk145815@ohio.edu (R.A.K.); rz537816@ohio.edu (R.L.Z.); et884814@ohio.edu (E.T.); gn454115@ohio.edu (G.G.N.); wiemels@ohio.edu (R.E.W.); 2 The Infectious and Tropical Disease Institute, Ohio University, Athens, OH 45701, USA

**Keywords:** PPIase, *S. aureus*, toxins, PpiB, PrsA, alpha-toxin, PSMs

## Abstract

Peptidyl-prolyl *cis/trans* isomerases (PPIases) are enzymes that catalyze the *cis*-to-*trans* isomerization around proline bonds, allowing proteins to fold into their correct confirmation. Previously, we identified two PPIase enzymes in *Staphylococcus aureus* (PpiB and PrsA) that are involved in the regulation of virulence determinants and have shown that PpiB contributes to *S. aureus* virulence in a murine abscess model of infection. Here, we further examine the role of these PPIases in *S. aureus* virulence and, in particular, their regulation of hemolytic toxins. Using murine abscess and systemic models of infection, we show that a *ppiB* mutant in a USA300 background is attenuated for virulence but that a *prsA* mutant is not. Deletion of the *ppiB* gene leads to decreased bacterial survival in macrophages and nasal epithelial cells, while there is no significant difference when *prsA* is deleted. Analysis of culture supernatants reveals that a *ppiB* mutant strain has reduced levels of the phenol-soluble modulins and that both *ppiB* and *prsA* mutants have reduced alpha-toxin activity. Finally, we perform immunoprecipitation to identify cellular targets of PpiB and PrsA. Results suggest a novel role for PpiB in *S. aureus* protein secretion. Collectively, our results demonstrate that PpiB and PrsA influence *S. aureus* toxins via distinct mechanisms, and that PpiB but not PrsA contributes to disease.

## 1. Introduction

*Staphylococcus aureus* is a Gram-positive bacterium that resides in the anterior nares of approximately one-third of the population. Diseases caused by *S. aureus* range in severity from minor skin and soft tissue infections, to life-threatening infections such as endocarditis, necrotizing fasciitis, and sepsis [1]. This incredible diversity in diseases is largely due to the multitude of virulence factors that *S. aureus* produces, such as exoenzymes that assist in the degradation of host molecules, adhesins that aid in attachment to surfaces, and toxins that lyse host cells [2,3,4,5]. Two of the best characterized toxins for their role in infection are α-toxin (encoded by the *hla* gene) and the phenol-soluble modulins (PSMs) [6,7].

Hla is a receptor-mediated pore-forming toxin, which binds the sheddase ADAM10 on the surface of host cells and disrupts their cellular membranes. Hla has been implicated as the primary toxin responsible for the lysis of rabbit erythrocytes, which have high amounts of ADAM10 coating their surface [6,8,9]. Interestingly, human red blood cells have very little ADAM10 on their surface and consequently, it takes high levels of accumulated Hla to lyse human erythrocytes. Lysis of human erythrocytes is more efficiently accomplished by the alpha phenol-soluble modulins (αPSMs) [10]. *S. aureus* encodes four αPSMs, each approximately 20–25 amino acids in size, on a single polycistronic transcript, called the αPSM transcript. A fifth αPSM (Hld or the delta-toxin) is encoded within the regulatory RNA molecule RNAIII. Studies on the regulation of both Hla and the αPSMs have been largely centered around the *agr* system. AgrA has been shown to bind directly to the promoter region of the αPSM transcript where it activates transcription, while *hla* translation is regulated by the *agr* effector RNAIII [11]. Interestingly, it has also been demonstrated that the αPSMs can regulate Hla production in murine skin and lung models of infection [12], although the exact mechanism is unclear. Investigating the regulation of these toxins will improve our understanding of their relative contribution during human infection and may help in the development of “anti-virulence” approaches to combat infections caused by *S. aureus*.

Peptidyl-prolyl *cis/trans* isomerases (PPIases) are a family of enzymes that catalyze the *cis*-to-*trans* isomerization of proline peptide bonds. Proline is a unique amino acid in that it can exist in both the *cis* and *trans* isomerization state in vivo. Correct protein folding is often not possible when a proline peptide bond is in the incorrect configuration and therefore the isomerization rate of proline peptide bonds can be the rate-limiting step in protein folding [13]. PPIase enzymes accelerate this isomerization and therefore assist in the regulation of proteins via a post-translational mechanism. Numerous studies have identified bacterial PPIases that contribute to virulence [14,15,16,17,18]. In addition to acting as foldases proteins, PPIases often have moonlighting roles and functions not limited to prolyl isomerization in the cell [19,20,21]. *S. aureus* encodes three PPIase enzymes—trigger factor (Tig), PrsA, and PpiB. Recent work in our lab (and others) has demonstrated that both PrsA and PpiB influence the activity of secreted virulence factors [21,22,23].

Initial work in our lab showed that a Δ*prsA* mutant has reduced phospholipase C (PI-PLC) activity and decreased protease activity, and that a Δ*ppiB* mutant has reduced hemolysis and nuclease activity [24]. A follow-up study demonstrated that PpiB contributes to virulence in a murine abscess model of infection, independently of its PPIase activity [21]. Recently, work by Lin et al. on the methicillin-sensitive *S. aureus* strain HG001, demonstrated that a Δ*prsA* mutant had better survival than wild-type (WT) in a murine systemic model of infection [22]. They also concluded that there was altered abundance of 67 exoproteins and 163 cell wall-associated proteins in a Δ*prsA* mutant, including the virulence factors surface protein A (SpA), immunodominant staphylococcal antigen B (IsaB) and the αPSMs.

Due to the contribution of these two PPIases to the regulation of multiple virulence determinants, we hypothesized that PrsA and PpiB would contribute to virulence in a murine systemic model of infection using a community acquired methicillin-resistant *S. aureus* (CA-MRSA) USA300 strain. In this study, we demonstrate that a USA300 Δ*ppiB* mutant is attenuated in both an abscess and systemic model of infection but there is no significant attenuation with a Δ*prsA* mutant. The same virulence trend was observed during intracellular survival assays using macrophage and human nasal-epithelial cells. To understand the molecular mechanism underlying the attenuation of virulence in the Δ*ppiB* mutant, we examined toxin production by measuring the hemolytic activity of culture supernatants against human and rabbit erythrocytes. We identify a significant reduction in the activity and secretion of the αPSMs in a Δ*ppiB* mutant as well as a reduction in the activity of Hla in both the Δ*prsA* and Δ*ppiB* mutants. Immunoprecipitation analysis of PpiB and PrsA suggests a role for PpiB in the Sec secretion pathway and that PrsA is involved in cell-wall processing. Together, these data suggest a role for two PPIase proteins in the regulation of *S. aureus* hemolytic toxins via two distinct mechanisms.

## 2. Results

### 2.1. PpiB Is Required for Virulence in a Murine Abscess and Systemic Model of Infection

Previously, we demonstrated that a Δ*ppiB* mutant was attenuated for virulence in an abscess model of infection using the CA-MRSA strain USA300 TCH1516 [21]. Recently, Lin et al. demonstrated increased survival of a Δ*prsA* mutant in a sepsis model using the methicillin-sensitive *S. aureus* strain HG001 [22]. To better understand the role of each PPIase protein, we performed both localized (abscess) and systemic (sepsis) murine infection models using the USA300 strain TCH1516 and isogenic PPIase deletion mutants. USA300 strains are the most frequent cause of community-acquired MRSA infection in North America and are also a frequent cause of hospital-associated MRSA (HA-MRSA).

**Figure 1:**
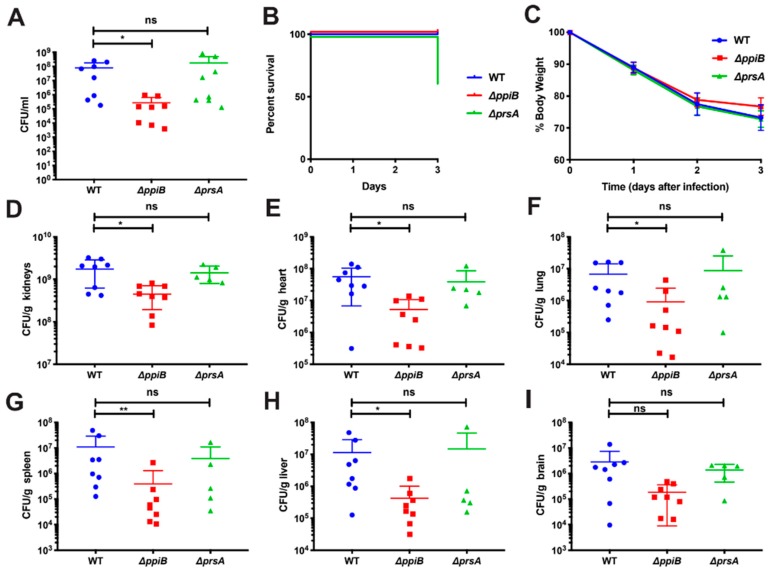
PpiB is required for virulence in a murine abscess (**A**) and systemic (**B**–**I**) model of infection. (**A**) Female 6-week-old BALB/c mice were injected subcutaneously with WT *S. aureus*, a Δ*ppiB* mutant or a Δ*prsA* mutant strain. The infection was allowed to proceed for 7 days. Mice were then sacrificed before abscesses were excised, homogenized, and diluted and plated to enumerate bacteria present in the abscesses. (**B**–**H**) Female 6-week-old BALB/c mice were injected retro orbitally with WT *S. aureus*, a Δ*ppiB* mutant or a Δ*prsA* mutant strain. The infection was allowed to proceed for 3 days. Mice were then sacrificed before organs were excised, weighed, diluted and plated to enumerate bacteria present in the organs. (**B**) Kaplan–Meyer curve for mouse survival during 3-day systemic infection. Three mice infected with the Δ*prsA* mutant strain died. (**C**) Percent initial body weight during 3-day systemic infection. The reduction in body weight in Δ*ppiB*-infected mice was less than that observed in WT or Δ*prsA*-infected mice, although the difference was not statistically significant. (**D**–**I**) CFU/g recovered from kidneys, heart, lung, spleen, liver, and brains of infected mice. In mice infected with the Δ*ppiB* mutant strain, a significant reduction in bacterial numbers was detected in the kidneys, heart, and lungs of infected mice. Experiments were performed with an *n* = 8 for each strain. Error bars represent standard deviation. Significance was determined by Student’s t test for the abscess model of infection (**A**). * *p* < 0.05; and by a Mann–Whitney *U*-test for systemic organ data, * *p* < 0.05.

The murine abscess model of infection was performed as described previously [25]. Briefly, 6-week-old BALB/c mice were injected in the lower right flank with 10^6^ CFU of either, W.T.; a Δ*ppiB* mutant or a Δ*prsA* mutant strain. Following a 7-day infection, animals were sacrificed, abscesses were excised and number of bacteria present was determined. The Δ*ppiB* mutant displayed a reduced bacterial load in a skin abscess, confirming our previous finding. However, a Δ*prsA* mutant showed no apparent alteration in recovered bacteria (Figure 1A).

We next performed a murine systemic model of infection as described by Alonzo et al. [26]. Six-week-old BALB/c mice were injected with 10^7^ CFU of each strain via the retro-orbital venous plexus and monitored for 3 days. Three of the eight Δ*prsA*-infected mice died over the course of infection (Figure 1B) and the surviving mice were monitored for change in weight (Figure 1C). Following 3 days of infection, mice were sacrificed, and the brain, lungs, heart, liver, spleen and kidneys were excised for processing. Each organ was homogenized, diluted, and plated to quantify the number of bacteria per organ. For Δ*ppiB*-infected mice, a significant reduction in bacterial burden was identified in the kidneys, heart, spleen, liver, and lungs (Figure 1D–I). A non-significant reduction in bacterial burden was also observed in the brain of the Δ*ppiB*-infected mice (*p* = 0.065), and overall a modest non-significant reduction in weight loss was also observed in the Δ*ppiB*-infected mice (Figure 1I,C). No difference in bacterial burden was observed in any of the organs from mice infected with the Δ*prsA* mutant (Figure 1D–I). This is in contrast to the findings of Lin et al. which showed increased murine survival when infected with a Δ*prsA* mutant [22].

### 2.2. PpiB is Required for Survival inside Macrophages and Human Nasal-Epithelial Cells

**Figure 2:**
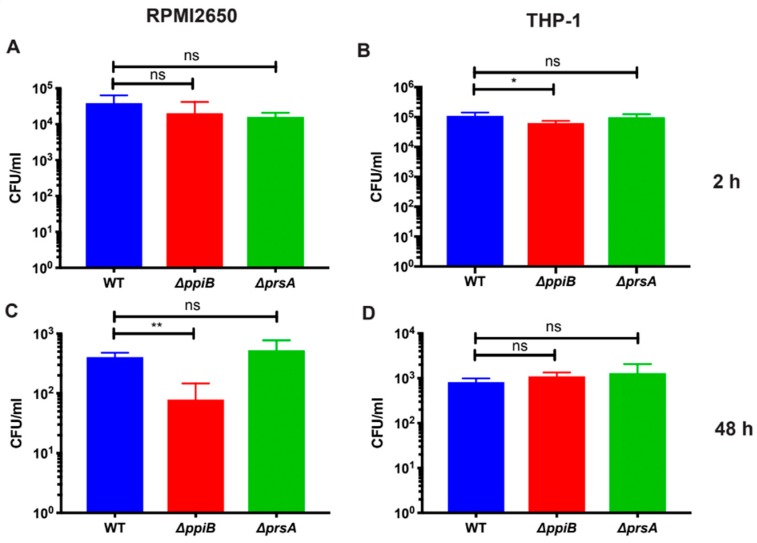
PpiB is required for survival in RPMI2650 nasal epithelial (**A**,**C**) and THP-1 macrophage cells (**B**,**D**). Gentamycin protection assays were performed at a multiplicity of infection (MOI) of 10 with WT, the Δ*ppiB* or the Δ*prsA* mutant strains. After 2 and 48 h of infection, bacterial cells were diluted and plated to enumerate surviving bacteria. Significance was determined by Student’s *t* test (A). * *p* < 0.05; ** *p* < 0.01.

Although long considered an extracellular pathogen, abundant evidence now exists to demonstrate that *S. aureus* has the ability to reside inside of both professional phagocytic cells and non-professional phagocytic cells during infection. To further characterize the contribution of PrsA and PpiB to virulence, we examined the ability of isogenic mutants to survive in the intracellular environment. These experiments were performed in professional phagocytic cells Tohoku Hospital Pediatrics (THP1) human monocytes/macrophage), and non-professional phagocytic cells Roswell Park Memorial Institute (RPMI) 2650, a human nasal epithelial cell line). Cells were infected at a multiplicity of infection (MOI) of 10 with either, W.T.; the Δ*ppiB* or the Δ*prsA* mutant strains. The three strains were used to infect THP-1 macrophages and RPMI 2650 cells and the number of intracellular bacteria determined at 2 h and 48 h. The Δ*ppiB* mutant displayed a small but significant decrease (*p* = 0.0365) in recovery compared to WT in macrophages at 2 h (Figure 2B). The Δ*ppiB* mutant also had a significant decrease (*p* = 0.0018) in recovery compared to WT in RPMI 2650 cells at 48 h (Figure 2C). These results are consistent with a Δ*ppiB* mutant being attenuated for virulence in murine models of infection. The Δ*prsA* mutant showed no significant differences in recovery at any of the selected timepoints in both cell types (Figure 2A–D).

### 2.3. Exoproteome Analysis Reveals Greater Alterations in Secreted Protein Abundance in a ΔppiB Mutant Than a ΔprsA Mutant

To investigate further why attenuation of virulence was observed in the Δ*ppiB* mutant but not the Δ*prsA* mutant, we analyzed the secreted proteome of the, W.T.; Δ*ppiB* and Δ*prsA* mutant strains to identify proteins with altered abundance that may contribute to infection. Secreted proteins were detected via label-free mass spectrometry and data was analyzed by the Scaffold program and compared based on average normalized abundance. Proteins with a minimum of a 2-fold change in abundance when compared to WT and a *p*-value < 0.05 (based on Student’s *t*-test) were considered for analysis. Based on these parameters, 86 proteins displayed altered abundance in culture supernatants of the Δ*ppiB* mutant (Table 1, Table S1). Sixteen were found in greater abundance in the Δ*ppiB* mutant, while 60 were less abundant compared to WT. In contrast, only 16 proteins were identified as having significantly altered abundance in the secreted fraction of a Δ*prsA* mutant compared to WT. Of these, 6 were found at a higher level and 10 were less abundant in culture supernatants of the Δ*prsA* mutant (Table 2, Table S2). Of note is that PpiB and PrsA peptides were detected in small amounts at 0.01 and 0.03, respectively, in their isogenic mutant strains when compared to WT. We attribute this to artifact based on the technique detecting small peptides and not full-length proteins.

**Figure 3:**
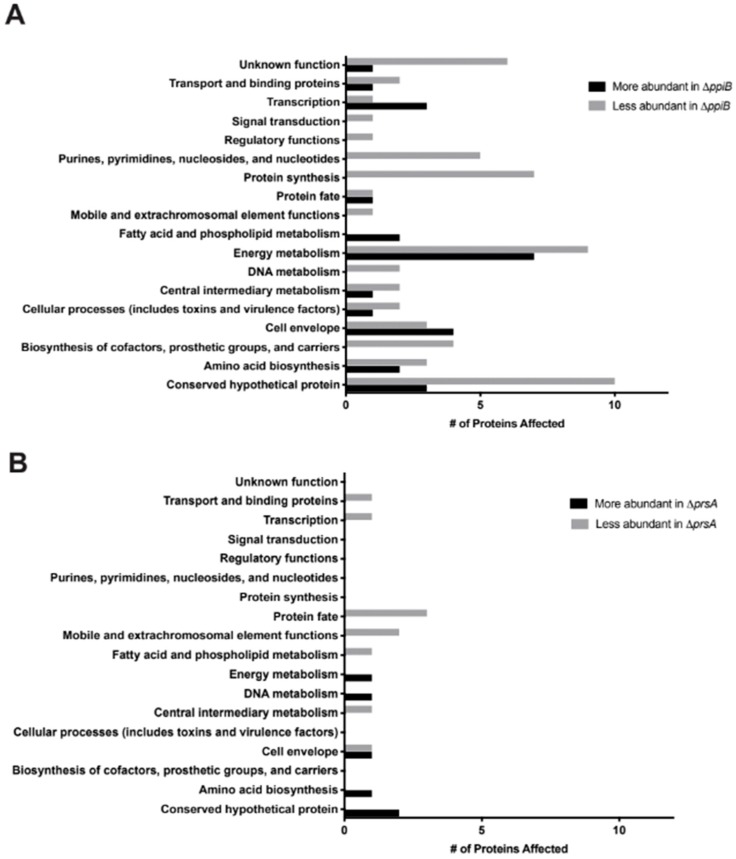
Exoproteome analysis reveals greater alterations in secreted protein abundance in a Δ*ppiB* mutant than a Δ*prsA* mutant. (**A**) Culture supernatants from a Δ*ppiB* mutant reveal that there are 86 proteins with altered abundance when *ppiB* is deleted. (**B**) Culture supernatants from a Δ*prsA* mutant reveal that there are 16 proteins with altered abundance when *prsA* is deleted. Proteins were grouped according to general function and the number of proteins falling into each functional category were plotted.

Proteins that made our cutoffs were grouped into categories based on known roles in the cell to identify common pathways or regulons with altered abundance (Figure 3). Notably, 2 of the 16 proteins with altered abundance in a Δ*prsA* mutant were cell envelope proteins. One of these, penicillin binding protein 2 (Pbp2a), was found in greater abundance in culture supernatants of the Δ*prsA* mutant. Work by Jousselin et al. demonstrated that *S. aureus* PrsA was involved in oxacillin resistance via regulation of *pbp2a*, and that a Δ*prsA* mutant had a reduced amount of Pbp2a in the cell wall [23]. We hypothesize that in the Δ*prsA* mutant, Pbp2a may have a defect in cell wall anchoring, which could account for the greater levels in the supernatant.

A large number of cytoplasmic proteins had decreased abundance in the exoproteome of a Δ*ppiB* mutant. Interestingly, we did not see the same trend in a Δ*prsA* mutant. Work by Wang et al. has recently demonstrated that *S. aureus* secretes numerous cytoplasmic proteins in extracellular vesicles to allow intracellular communication [27]. Notably, they show that this secretion is mediated by the αPSMs, which promote the release of extracellular vesicles from the bacterial cell. This finding, coupled with our previous work that shows a defect in human erythrocyte lysis in a Δ*ppiB* mutant [21,24], led us to hypothesize that PpiB might be regulating secretion of the αPSMs. A reduction in the amount of αPSMs could explain why cytoplasmic proteins were found in less abundance in a Δ*ppiB* mutant.

### 2.4. A ΔppiB Mutant Has Reduced PSM Production

**Figure 4:**
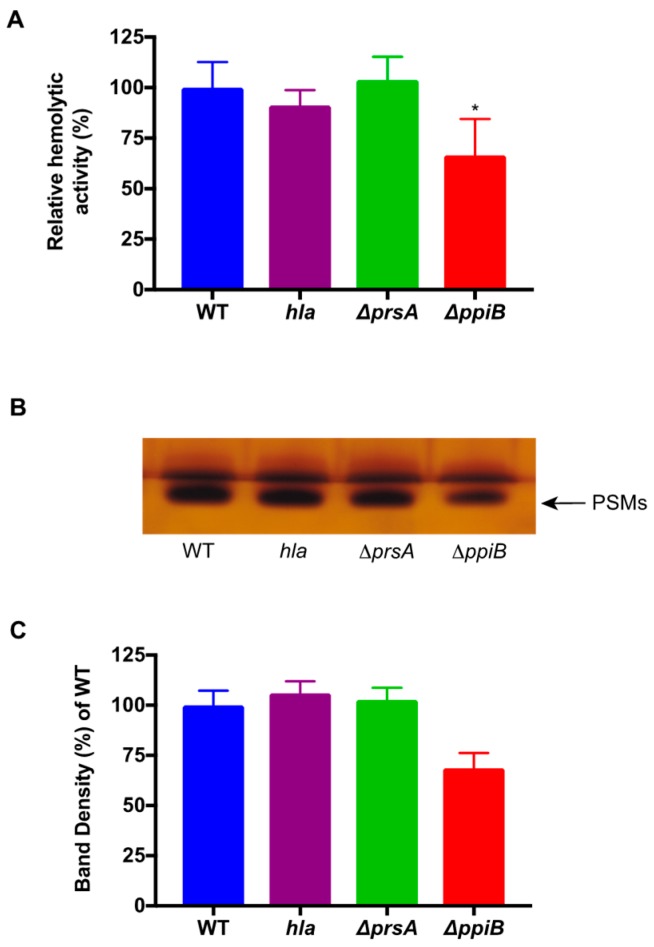
Decreased αPSM production in a Δ*ppiB* mutant. (**A**) Hemolytic activity of butanol-extracted peptides. Butanol-extracted samples were resuspended in water and incubated with whole human blood. The deletion of *ppiB* results in a significant decrease in hemolytic activity in comparison to WT. (**B**) SDS-PAGE analysis of butanol-extracted samples from panel A. PSM levels are reduced in a Δ*ppiB* mutant. (**C**) Densitometry analysis of PSMs on SDS-PAGE gel. Densitometry analysis was performed on duplicate samples and normalized to WT. The PSM band showed decreased abundance in the *ppiB* mutant strain (68.5% of WT). No differences in PSM abundance were observed in the *hla* or *prsA* mutant strains. Significance was determined by Student’s *t* test. * *p* < 0.05.

Our previous publications demonstrated that culture supernatants from a Δ*ppiB* mutant were less hemolytic for human blood than supernatants from the WT [21,24]. Although the reduction in hemolytic activity was clear, the specific toxin responsible for this phenotype was not identified. Based on the results of the exoproteome analysis above, and the facts that the primary toxins responsible for human erythrocyte lysis are the αPSMs and that Hla activity against human erythrocytes has been shown to be negligible (Figure S1, [28]), we hypothesized that there is decreased abundance (or activity) of αPSMs in a Δ*ppiB* mutant. To test this hypothesis, we performed a butanol extraction to isolate the αPSM peptides from *S. aureus* culture supernatants [29]. This experiment allows visualization of lysis mediated exclusively via the PSMs, as they are the only toxin that will be extracted from culture supernatants, and thus, no other hemolysins will be present. The resulting extracted peptides were used in a hemolysis assay and analyzed by SDS-PAGE to visualize peptide abundance (Figure 4). Extracts from a Δ*ppiB* mutant displayed a modest (1.5-fold), yet significant reduction in hemolysis when compared to WT (Figure 4A). No reduction in hemolytic activity was observed in peptide extracts taken from the Δ*prsA* mutant or a *hla* mutant (negative control). SDS-PAGE analysis of extracts revealed a band corresponding to the size to the PSM peptides in each of the samples that was reduced in a Δ*ppiB* mutant (Figure 4B). Densitometry analysis was then performed on the SDS-PAGE gel with duplicate samples. Densitometry values were determined using Image J and normalized to WT (Figure 4C). Results show reduced PSM abundance in the Δ*ppiB* mutant (68.5% compared to the WT), mirroring the decrease in PSM activity observed in Figure 4A. Together, these results demonstrate a reduction in PSM abundance when *ppiB* is absent from the cell, confirming that PpiB influences PSM production.

### 2.5. The ΔppiB and ΔprsA Mutants Both Display Reduced Hemolysis of Rabbit Erythrocytes

**Figure 5:**
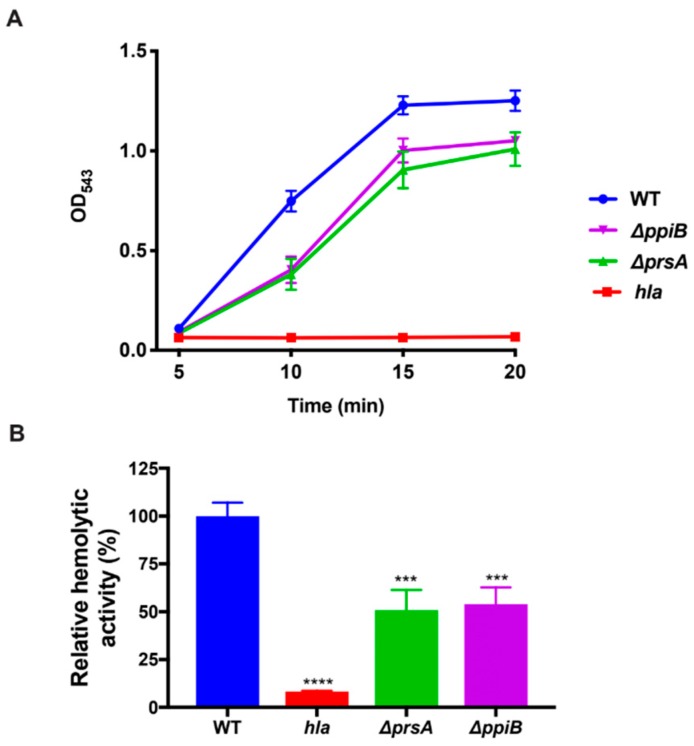
A *ppiB* and *prsA* mutant display reduced hemolysis in rabbit blood. Rabbit erythrocyte lysis assays were performed using *S. aureus* culture supernatants and whole rabbit blood. (**A**) A decrease in hemolytic activity against rabbit erythrocytes over time was observed using culture supernatants from *ppiB*, *prsA* and *hla* mutant strains. (**B**) A representative time point at 10 min reveals a significant reduction in hemolysis in the *ppiB* and *prsA* mutant strains. The data presented in A and B are the averages of four replicates. Significance was determined by Student’s t test. ****, p < 0.001; ***, p < 0.005.

The αPSMs have been shown to regulate Hla by delaying transcription and subsequently, Hla secretion [12]. Consequently, we hypothesized that alpha-toxin activity would also be altered in a Δ*ppiB* mutant. Previously, we demonstrated that the Δ*ppiB* mutant displayed a slight reduction in hemolytic activity against rabbit erythrocytes, however, results were not deemed significant [21]. To better test the activity of Hla in a Δ*ppiB* mutant, we repeated the hemolysis assay over a time course of 20 min, with measurements taken every 5 min to determine the degree of lysis. Interestingly, culture supernatants from both a Δ*ppiB* and a Δ*prsA* mutant displayed reduced hemolysis at earlier time points (Figure 5A) when compared to WT. A representative time point at 10 min displayed a significant reduction in rabbit erythrocyte lysis in both the Δ*ppiB* and Δ*prsA* mutants, although not to the levels of a *hla* mutant strain (Figure 5B). At later timepoints (>20 min), the degree of hemolytic activity in the Δ*ppiB* and Δ*prsA* mutant samples increased to a level similar to that of the WT. This result explains our previous observation of a modest, non-significant reduction in hemolytic activity in the Δ*ppiB* mutant [21].

Taken collectively, these data suggest that both PpiB and PrsA are involved in the regulation of Hla. To investigate if this regulation occurred at the level of transcription or post-trancriptionally, RT-qPCR was performed to examine *hla* and αPSM transcript levels in the, W.T.; Δ*ppiB* and Δ*prsA* strains. No significant differences were detected in either transcript, indicating that the regulation is likely post transcriptional (Figure S2). We postulate that the reduction in Hla in a Δ*ppiB* mutant could be due to the reduction of αPSMs in culture supernatants. This supports the findings of Berube et al. in which they demonstrate that the αPSMs regulate Hla production during infection [12]. Since no significant reduction in PSM activity was observed in a Δ*prsA* mutant, we hypothesize that PrsA is regulating Hla independently of the PSMs. One explanation for why a Δ*prsA* mutant may have reduced cytolytic activity against rabbit erythrocytes is if the PPIase activity of PrsA is required to help assist in the folding of Hla. If this is the case, deleting *prsA* could lead to less active Hla in culture supernatants.

### 2.6. In Vivo Immunoprecipitation Identifies Greater Abundance of PpiB Target Proteins Than PrsA

Previous work in our lab has demonstrated that PpiB contributes to *S. aureus* virulence independently of its PPIase activity, suggesting an alternative function/activity for this protein [21]. To further investigate this alternative function, and to elucidate how PpiB and PrsA independently regulate *S. aureus* toxins, we performed an immunoprecipitation experiment to identify proteins that interact with PpiB and PrsA. The *ppiB* gene was amplified, HA-tagged, and cloned into the multicopy plasmid pMK4 under the control of its native promoter (plasmid pRKC0131). To identify proteins interacting with PpiB, pRKC0131 (*ppiB*-HA) was transduced into the Δ*ppiB* mutant strain, and immunoprecipitation performed using anti-HA magnetic beads. Immunoprecipitation experiments were performed by formaldehyde crosslinking proteins in cultures that had been grown for 3 h to mid-exponential phase. A negative control strain containing the empty pMK4 vector in the Δ*ppiB* mutant was used for comparison. Magnetic beads containing PpiB-HA (and associated proteins) were collected and analyzed via mass-spectrometry to identify all peptides associated with PpiB. A similar analysis was performed for PrsA using a His-tagged *prsA* construct in the same pMK4 plasmid (plasmid pRKC0126). Anti-His magnetic beads were analyzed and compared to an empty vector control in the Δ*prsA* mutant strain.

Data were analyzed using the Scaffold program and compared via the exclusive spectrum count of each protein identified in the samples. Proteins with a minimum of 3-fold increase in abundance (in the tagged strain compared to the empty vector control) were selected for further analysis. We also excluded any data where the average spectrum count was >10 for both the overexpresser and empty vector strains. Surprisingly, neither the αPSMs nor Hla were identified in the immunoprecipitation assay, suggesting that the role of PpiB and PrsA in regulating these toxins is indirect (see below).

As predicted (based on its cellular location), PrsA was found to interact with a large number of cell-wall anchored proteins (Table 3, Table S3). This included the signal peptidase I (SpsB) which assists in the anchoring of cell-wall proteins by cleaving the N-terminal signal peptide in the general secretory pathway. Work by Schallenberger et al. demonstrated that inhibition of SpsB with arylomycin led to increased abundance of the proteins PrsA, HtrA and SAOUHSC_01761 [30]. They suggested the reason for increased secretion of these proteins could have been as a response to the inhibition of SpsB (28). Notably, both *htrA* and SAUSA300_1606 (SAOUHSC_01761 homologue in TCH1516) both have altered abundance in the secreted fraction of a Δ*prsA* mutant (Table. 3, Table S3). HtrA is a highly conserved protein in Gram-positives where it functions as a chaperone and a protease [31,32]. SAUSA300_1606 is an uncharacterized protein and relatively little is known about it other than its induction during vancomycin treatment [33]. How these proteins might be interacting in the cell remains unknown, but our findings support a connection between the anchoring of PrsA to the cell wall and its connection to antibiotic resistance.

The largest fold enrichment for a protein identified in the PpiB immunoprecipitation analysis (after PpiB itself) was for the Sec translocase subunit SecA1 (8.5-fold enrichment, Table 4, Table S4). SecA is the ATPase that facilitates protein translocation through the SecYEG translocon, in the general secretory pathway [34]. In *Escherichia coli*, the chaperone protein SecB transports newly synthesized proteins from the ribosome to SecA for secretion [35]. Interestingly, *S. aureus* does not encode a SecB homologoue, and to date it is unknown if any alternative chaperone substitutes for SecB in the secretion process [34]. As previously stated, many PPIases also function as chaperone proteins [36,37,38]. This, in addition to our recent finding that PpiB possesses a PPIase-independent activity [21], and the data shown here of an association between PpiB and SecA, suggests that PpiB may function as a chaperone in the general secretion system. If so, this could account for the reduced activity of secreted virulence factors, such as Hla and nuclease (Nuc), in a Δ*ppiB* mutant [24].

## 3. Discussion

**Figure 6:**
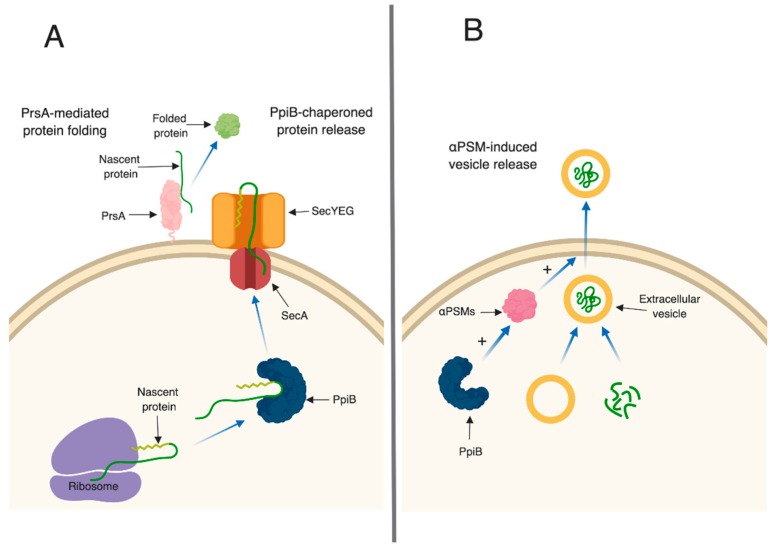
Schematic diagram of proposed mechanisms of PpiB and PrsA. (**A**) PpiB functioning as a chaperone to deliver nascent proteins to the Sec secretion machinery. Once secreted, proteins begin to fold and the membrane-anchored PrsA, which acts as a PPIase, assists this process. (**B**) PpiB positively regulates the αPSMs. When active αPSMs are within the cell, they promote the release of EVs from the membrane. Image created with BioRender.

In bacteria, PPIase enzymes are traditionally studied for their roles in protein (re)folding. In Gram-positive bacteria, parvulin-type PPIases (including PrsA) are often anchored on the external leaflet of the cell membrane, and are thought to fold secreted proteins after they have been translocated out of the cell. *S. aureus* PrsA appears to function in a similar manner. A *prsA* mutant strain has decreased proteolytic and phospholipase activity [24], increased sensitivity to β-lactam antibiotics [23] and decreased hemolytic activity (this study). We hypothesize that all of these defects arise as a result of defective protein folding in the absence of PrsA. This idea is supported by a number of observations in our proteomic and immunoprecipitation data analysis. First, the number of secreted proteins displaying altered levels in *prsA* mutant culture supernatants was relatively low (16 proteins, Table 2, Table S2). This suggests that PrsA targets are present in these supernatants, however, they may exhibit reduced activity due to incorrect folding (Figure 6A). Second, the majority of proteins found to interact with PrsA by immunoprecipitation are localized to the cell envelope and play important roles in cell wall synthesis and stability. Of particular interest was the identification of Pbp2a in the immunoprecipitation assay. This result supports the findings of Jousselin et al. and validates the approach used in this study to identify proteins that interact with PrsA [23]. Interestingly, EsaA, a component of the recently identified *S. aureus* type 7 secretion system, was identified in the immunoprecipitation assay as interacting with PrsA and was also found at lower levels in the culture supernatant. This may indicate a potential role for PrsA in type 7 secretion.

An unexpected finding in this study was that PrsA did not contribute to virulence in either a murine abscess or sepsis model of infection. This result conflicts with the results obtained by Lin et al., who demonstrate attenuation of virulence in a HG001 *prsA* mutant [22]. One possible explanation for the discrepancy in results observed is the strain background used in each study. HG001, the strain used by Lin et al., is a methicillin-sensitive strain derived from NCTC8325 (with the *rsbU* gene repaired) [22]. The strain used in this study, TCH1516, is a methicillin-resistant USA300 strain. USA300 strains typically produce high levels of toxins and previously it was shown that a USA300 strain is more hemolytic than HG001 [39]. We speculate that, in USA300 strains, the high level of toxin production and overlapping activities of *S. aureus* toxins may negate the requirement for PrsA activity when it comes to causing disease in a mouse model of infection. In strains with relatively lower levels of toxin production (such as HG001), the loss of PrsA activity may have a more dramatic effect and result in attenuation. To test this hypothesis, we are currently broadening our investigations and exploring the role of PrsA in a variety of *S. aureus* strains.

While the role of PrsA in *S. aureus* appears to be similar to that of homologues in other Gram-positive bacteria (such as *Listeria monocytogenes*), the mechanism of action of PpiB remains elusive. PpiB clearly plays an important role in infection, as a *ppiB* mutant is attenuated in abscess and sepsis models of infection, and displays reduced intracellular survival. The activity and abundance of several virulence factors (including nuclease, alpha toxin, and the PSMs) is reduced in *ppiB* culture supernatants, which explains the attenuation of virulence, however the mechanism through which PpiB functions is not clear. We previously demonstrated that PpiB (i) is found in the cytoplasm, (ii) possesses PPIase activity, and (iii) its PPIase activity was dispensable during infection [21,24]. Taken together, this implies that PpiB functions inside the cell, in a PPIase-independent manner, to influence virulence factor production. Our first indication of a potential biological role for PpiB comes from the secreted proteomic and immunoprecipitation data presented herein. SecA, a component of the general secretion pathway in *S. aureus*, was found to interact with PpiB and was also found at significantly reduced levels in *ppiB* mutant culture supernatants. In *E. coli*, during general secretion, a chaperone protein called SecB binds to newly synthesized proteins and delivers them to SecA for translocation. No SecB homologue has been identified in *S. aureus*, therefore, based on our data, we hypothesize that one potential role for PpiB could be to functionally compensate for SecB and chaperone proteins prior to secretion (Figure 6A). Thus, the loss of PpiB could result in general secretion defects and/or the secretion of misfolded proteins. This might explain why so many proteins had altered abundance in the culture supernatants of *ppiB* mutant strains compared to *prsA* mutants (86 proteins altered in *ppiB* mutant, 16 in *prsA* mutant). Another role of chaperone proteins is to prevent the aggregation of misfolded proteins under stress [40]. If PpiB is functioning as a chaperone, it could additionally function to protect the αPSM peptides within the *S. aureus* cell. If this is the case, it could explain why there are less active αPSMs in culture supernatants of a Δ*ppiB* mutant.

*S. aureus*, like many other cells, has the ability to produce and secrete extracellular vesicles (EVs) [27]. Recently, the formation of EVs in *S. aureus* has been shown to be enhanced by the αPSMs [27]. EVs are loaded with proteins, including many proteins typically thought of as being cytoplasmic. The presence of EVs in culture supernatants may explain why cytoplasmic proteins are commonly identified during proteomic studies of secreted fractions, which we also observed in our proteomic data set. Interestingly, of the 86 proteins that were altered in abundance in *ppiB* mutant supernatants, a large proportion of them are considered cytoplasmic. These proteins were present in WT supernatants, but decreased in *ppiB* mutant supernatants. It is possible that PpiB plays a role in EV biogenesis and that in the mutant there is reduced EV production, which accounts for the variation in cytoplasmic proteins. Since a *ppiB* mutant produces less αPSMs which are necessary for EV release, it is also possible that the same number of EVs are being generated in a Δ*ppiB* mutant but they are unable to be released from the cell due to the decrease in αPSMs (Figure 6B). We are currently investigating EV production in a *ppiB* mutant.

In conclusion, while the role of PpiB in pathogenesis is clear, its molecular mechanism of action remains uncertain. The results generated in this study further our understanding of *S. aureus* virulence factors regulated by PpiB and suggest that PpiB may be functioning in a secretion-related manner. Further studies are being conducted to investigate this novel protein to better understand how it regulates virulence factor production and virulence.

## 4. Materials and Methods

### 4.1. Strains and Strain Construction

All bacterial strains and plasmids are listed in Table 5 and oligonucleotides in Table 6. The Δ*ppiB* and Δ*prsA* mutant strains were constructed via allelic exchange as previously described [21,24,41]. For overexpresser strains used in the immunoprecipitation, plasmids pRKC0131, pRKC0126 and pMK4 (empty vector) were transduced into RKC0323 (Δ*ppiB*) and RKC0085 (Δ*prsA*) respectively. A Δ*αpsm* strain was constructed by allelic exchange using plasmid pJB38 [42]. DNA sequences flanking the αPSM transcript were amplified using primer pairs 490/644 and 645/493 and a third sequence containing the erythromycin cassette from the *bursa aurealis* transposon was amplified using primers 638 and 639 [42]. These three fragments were cloned to generate plasmid pRKC0674. This plasmid was recombined onto the *S. aureus* chromosome and excised to make the deletion strain according to previously published protocol [41].

### 4.2. Bacterial Growth Conditions

*S. aureus* cultures were grown in tryptic soy broth (TSB) shaking at 37 °C. Where appropriate, the antibiotic chloramphenicol was used at the concentration of 5 μg mL^−1^. For analysis of culture supernatants, *S. aureus* cultures were synchronized as follows. Replicate overnight cultures were grown in 5 mL of TSB for 15 h. The next day, cultures were diluted 1:100 in 10 mL of pre-warmed TSB and grown for 3 h to mid-exponential phase. The 3-h cultures were then diluted into 25 mL of TSB in 250 flasks and normalized to an optical density at 600 nm (OD_600_) of 0.05. Resulting flasks were grown overnight for 15 h.

### 4.3. Murine Abscess Model of Infection

A subcutaneous abscess infection was performed as described by us previously [21]. Cultures were grown for 2.5 h in TSB to an OD_600_ of 0.75. Resulting bacterial cells were pelleted via centrifugation and resuspended in sterile phosphate-buffered saline (PBS) to prepare an inocula of 10^6^ CFU/50 μL. Each inoculum was then confirmed via serial diluting and plating. Six-week-old female BALB/c mice were anesthetized by isoflourane inhalation before being shaved at the right flank and treated with Nair to remove fur. Mice were then injected with 50 μL of their respective strains and the infection was allowed to proceed for 7 days. Following 7 days of infection, mice were euthanized with CO_2_ and abscesses were excised and homogenized. Homogenates were serial diluted and plated onto TSB to count recovered CFU/ abscess.

### 4.4. Murine Systemic Model of Infection

A systemic model of dissemination was chosen to mimic septic infection and cultures were prepared as previously described by Spaan et al. [49]. Cultures were grown for approximately 2.5 h in TSB to an OD_600_ of 0.75. Resulting bacterial cells were pelleted via centrifugation and resuspended in sterile phosphate-buffered saline (PBS) to prepare an inocula of 10^7^ CFU/100 μL. Six-week-old BALB/c mice were injected retro-orbitally with 100 μL of bacterial cultures and the infection was allowed to proceed for 3 days. Following 3 days of infection, mice were euthanized with CO_2_ and the brain, lungs, heart, liver, kidneys and spleen were harvested. Each organ was weighed and homogenized before serial dilution and plating was conducted to quantify recovered bacteria/ organ.

### 4.5. Macrophage Infection and Cell Differentiation

THP-1 infection assays were performed as outlined in Carroll et al. [50]. Macrophages were seeded at a density of 2 × 10^5^ macrophages/well in a volume of 500 µL of RPMI 1640 with 10% FBS and 1% penicillin/streptomycin. A multiplicity of infection (MOI) of 10 was used to infect each well (2 × 10^6^ bacteria/well). Strains were prepared by taking 250 µL of overnight culture grown at 37 °C and inoculating it into a 250 mL flask containing 25 mL TSB. The bacteria were then grown in a shaking 37 °C incubator until their optical density (OD_600_) reached 0.4–0.6. The volume of bacteria needed to perform the infection was then transferred into a 1.5 mL centrifuge tube and pelleted for 20 min at 3000 rpm. The supernatant was removed and discarded. The pellet was then washed with 500 µL of phosphate buffered saline (PBS) and pelleted for 20 min at 3000 rpm. The pellet was resuspended in 20% human serum and 80% PBS. This solution was then incubated in a 37 °C water bath for 30 min to opsonize the bacteria. Opsonized bacteria were resuspended into the correct volume of 1640 RPMI medium with 10% FBS.

The macrophages were washed twice with 37 °C prewarmed PBS. Each well received 500 µL of the opsonized bacteria resuspended in 1640 RPMI with 10% FBS. Plates were centrifuged at 1000 rpm for 10 min at room temperature and placed into a 37 °C incubator supplemented with 5% CO_2_ for 60 min. After 60 min, the medium was aspirated, and the wells were washed once with prewarmed PBS as described above. To each well, 500 µL of RPMI 1640 with 10% FBS and 30 µg/mL of gentamicin was added. Cells were incubated in a 37 °C incubator supplemented with 5% CO_2_ for 60 min. The end of this 60 min incubation marked hour 0 of intracellular. After this incubation, the medium was aspirated and replaced with 500 µL of RPMI 1640 with 10% FBS and 5 µg/mL of gentamicin for the rest of the infection.

Intracellular bacteria were harvested from the macrophages at 2 h and 48 h, representing initial and late stages of infection. Medium was aspirated, and the cells were washed twice with prewarmed PBS as described above. A 500 µL aliquot of 0.5% Triton X-100 in PBS was added to each well to lyse the macrophages and release the intracellular bacteria. The lysates for each time point were serially diluted in PBS in sterile 96-well plates. A volume of 100 µL of each of the diluted lysate was plated on TSA plates. Plates were incubated in a 37 °C incubator and colonies were counted to determine the number of recovered intracellular bacteria and calculated to determine CFU/mL.

### 4.6. Nasal Epithelial Cell Infection

Strains were prepared and washed in the same way as described above except the bacteria were not opsonized. After washing the bacterial pellet with PBS, the bacteria were resuspended into the correct volume of EMEM with 10% FBS. RPMI 2650 cells were seeded at the same density and volume as described above except using EMEM with 10% FBS and 1% penicillin/streptomycin. Cells were washed twice with prewarmed PBS and infected with bacteria at an MOI of 10 (2 x 10^6^ bacteria/well).

### 4.7. Exoproteome Analysis

Bacterial strains were grown in triplicate in 5 mL overnight cultures shaking at 37 °C. The next day, cultures were diluted 1:100 in 10 mL of TSB and incubated for 3 h shaking at 37 °C. After 3 h, the OD_600_ of each culture was measured and each sample was normalized to an OD_600_ of 0.05 in 100 mL of TSB. After 15 h of growth in the flask, samples were split into 2 × 50 mL tubes and centrifuged at 3000 rpm for 15 min. Cell-free supernatants were harvested and filter sterilized through a 0.45 μm filter disk to ensure all bacterial cells were removed from the sample. An amount of 10 mL of TCA was added to the culture supernatants and incubated overnight at 4 °C. The following day, samples were centrifuged at 11,000 RPM for 10 min and the supernatant was removed. Resulting pellets containing concentrated proteins were washed with ice-cold acetone and sent to the University of Nebraska Lincoln Proteomics Facility for mass spectrometry analysis.

TCA precipitated samples were dissolved in a solution of 7M urea, 2M thiourea, 5 mM DTT, 0.1 M Tris, pH 8 and 1× PhosStop by gentle shaking for 1.5 h at 24 °C. After reduction, the samples were alkylated for 30 min using a 3-fold molar excess of iodoacetamide. The protein concentration was determined using the CB-X protein assay kit. An amount of 100 μg of protein in 10 μL of the urea solution was diluted and digested with 2 μg trypsin (1:50 enzyme:substrate ratio) for 16 h overnight, then an additional 1 μg of trypsin was added and digestion continued for a further 4 h. An amount of 200 ng of each of the 9 samples was run by nanoLC-MS/MS using a 2 h gradient on a 0.075 mm × 250 mm C18 Waters CSH column feeding into a Q-Exactive HF mass spectrometer.

Raw datafiles were loaded directly in to Progenesis QI for proteomics version 2.0 and alignment of the chromatograms showed a ≥95% match. Features (peaks) were extracted across all runs and areas under the peaks calculated. Runs were normalized using the “normalize to all proteins” setting. Data were exported from Progenesis and analyzed using the search engine Mascot (Matrix Science, London, UK; version 2.6.1, 2016. Mascot was set up to search 2 databases: the common contaminants database cRAP_20150130 database (117 entries) and a combined database containing 2 uniprot reference proteomes for *S. aureus* NCTC8325 & C0673_20170607 (5705 entries) assuming the digestion enzyme trypsin. Mascot was searched with a fragment ion mass tolerance of 0.060 Da and a parent ion tolerance of 10.0 PPM. Oxidation of methionine; deamidated of asparagine and glutamine; and carbamidomethyl of cysteine were specified in Mascot as variable modifications.

### 4.8. Butanol Extraction of PSMs and Densitometry Analysis

PSM peptides were isolated as previously described [29]. An amount of 5 mL of synchronized, cell-free culture supernatants was incubated shaking at 37 °C with 3 mL of *n*-butanol for 2 h. Cultures were then centrifuged and 1 mL of the organic layer was removed for vacuum centrifugation for 12 h at 5000 rpm. Samples containing the PSMs were then resuspended in water and utilized for human erythrocyte hemolysis assays. The same set of samples was run on SDS-PAGE gels in duplicate and silver stained. Densitometry analysis of the duplicate extract samples was performed using Image J software. Samples were averaged and normalized to the percent density of WT.

### 4.9. Human-Erythrocyte Hemolysis Assay

Synchronized bacterial cultures were grown in quadruplicate as described in bacterial growth conditions for 15 h. Samples were centrifuged and cell-free culture supernatants were harvested and diluted 1:2 in reaction buffer containing 40 mM CaCl_2_, and 1.7% NaCl. An amount of 25 µL of whole-human blood was added to samples and they were incubated at 37 °C while rotating. After 10 min, samples were centrifuged at 5500× *g* and the resulting supernatant was transferred to a 96-well plate. The degree of erythrocyte lysis was determined by reading sample absorbance at an OD_543_.

### 4.10. Rabbit Erythrocyte Hemolysis Assay

Bacterial strains were grown in quadruplicate in a 25 mL flask of TSB as described above. Cultures were then centrifuged to obtain the cell-free supernatant. Supernatants were diluted 1:10 before being further diluted 1:2 in reaction buffer containing 40 mM CaCl_2_, and 1.7% NaCl and incubated in a 37 °C water bath with 125 µL of rabbit blood. An amount of 200 µL of samples was removed every 5 min after incubation and centrifuged at 5500× *g*. An amount of 100 µl of supernatant was transferred to a 96-well plate and erythrocyte lysis was determined by reading the absorbance of the samples at OD_543_.

### 4.11. Protein Immunoprecipitation Assay

Duplicate bacterial cultures were inoculated into 100 mL of TSB with appropriate antibiotics in 250 mL flasks for 3 h to mid-exponential phase. Samples were centrifuged at 3000 rpm for 20 min and pellets were resuspended in sterile PBS. In total, 37% formaldehyde was added to cultures to a final concentration of 1% and a 20 min shaking incubation was performed at room temperature. The reaction was quenched with the addition of glycine to a final concentration of 200 mM. Cells were centrifuged and the remaining pellets were washed with sterile PBS and then re-suspended in sterile water. Cells were then lysed with lysostaphin at a concentration of 10 mg/mL and incubated at 37 °C for 30 min. Following this, cells were treated with DNase I and incubated at 37 °C for 30 min. Resulting lysates were then sonicated at 20% amplitude, 2 × 15 s. Samples were then centrifuged at 13,000 rpm for 1 min and supernatants were used for immunoprecipitation.

For immunoprecipitation, anti-HA or anti-His magnetic beads were added to respective samples and incubated at 4 °C for 1 h. Beads were collected with a magnetic rack and washed 10 times with 1× PBS. Final washed beads were resuspended in 20 µL of water and sent to the University of Nebraska Lincoln Proteomics Facility for analysis.

The 2 magnetic bead samples were resuspended in ammonium bicarbonate containing 5 mM DTT and reduced at 37 °C for 1 h. Samples were then alkylated (10 mM IAM for 20 min at 22 °C in the dark). The IAM was quenched with a molar equivalent of DTT. Trypsin was added and digestion carried out overnight at 37 °C. The digest was dried down and dissolved in 2.5% acetonitrile, 0.1% formic acid. An amount of 5 uL of one digest was run by nanoLC-MS/MS using a 2 h gradient on a 0.075 mm × 250 mm C18 Waters CSH column feeding into a Q-Exactive HF mass spectrometer.

All MS/MS samples were analyzed using Mascot (Matrix Science, London, UK; version 2.6.1, 2016). Mascot was set up to search the cRAP_20150130.fasta (117 entries); uniprot_S_aureus_USA300_20170823 database (2607 entries) assuming the digestion enzyme trypsin. Mascot was searched with a fragment ion mass tolerance of 0.060 Da and a parent ion tolerance of 10.0 PPM. Deamidated of asparagine and glutamine, oxidation of methionine and carbamidomethyl of cysteine were specified in Mascot as variable modifications.

Scaffold (Proteome Software Inc., Portland, OR, USA, version Scaffold_4.8.4, 2017) was used to validate MS/MS-based peptide and protein identifications. Peptide identifications were accepted if they could be established at greater than 80.0% probability by the Peptide Prophet algorithm [51] with Scaffold delta-mass correction. Protein identifications were accepted if they could be established at greater than 99.0% probability and contained at least 2 identified peptides. Protein probabilities were assigned by the Protein Prophet algorithm [52]. Proteins that contained similar peptides and could not be differentiated based on MS/MS analysis alone were grouped to satisfy the principles of parsimony. Proteins sharing significant peptide evidence were grouped into clusters.

### 4.12. Reverse Transcriptase-Quantitative PCR (RT-qPCR)

RT-qPCR was performed as described previously [47]. Briefly, bacterial pellets were collected 6 h after subculture and total RNA was isolated. Complimentary DNA (cDNA) was synthesized from 1 µg of total RNA using iScript reverse transcriptase (Bio-Rad) according to the manufacturer’s directions. The cDNA was diluted 10 times and used in SYBR Green reactions in technical duplicates to analyze the expression of *αPSM*, and *hla.* Transcription of the housekeeping gene *gyrB* was used as the endogenous control in each strain. Relative expression was determined by first comparing the amount of each individual gene transcript to *gyrB* within the same strain, followed by expression of these values in comparison to each respective gene in WT strain TCH1516.

### 4.13. Ethics Statement

Whole human-blood was isolated from donors in agreement with the Ohio University Institutional Review Board (Identification code; 17-X-79 date of approval: 22 February 2019). Rabbit blood was purchased from Hemostat-laboratories. Six-week-old female BALB/c mice were purchased from Envigo and held at the Ohio University Office of Laboratory Animal Resources. Animal work was performed under approval of the Institutional Animal Care and Use Committee (Identification code; 18-H-029 date of approval: 04 September 2018) at Ohio University and performed by trained lab personnel.

## Figures and Tables

**Table 1:** Proteins with altered abundance in Δ*ppiB* culture supernatants.

Gene Designation	Protein Name	Fold Change ^1^	Functional Grouping
SAUSA300_1533	YdfA	25.51	Conserved hypothetical protein
SAUSA300_0062	ArcB	23.74	Amino acid biosynthesis
SAUSA300_0962	QoxB	18.74	Energy metabolism
SAUSA300_0226	FadB	13.36	Fatty acid and phospholipid metabolism
SAUSA300_2581	SasA	7.36	Cell envelope
SAUSA300_1305	OdhB	4.93	Energy metabolism
SAUSA300_0912	FabI	4.78	Fatty acid and phospholipid metabolism
SAUSA300_1594	YajC	4.71	Protein fate
SAUSA300_2061	AtpH	3.70	Energy metabolism
SAUSA300_2060	AtpA	3.44	Energy metabolism
SAUSA300_0547	SdrD	3.37	Cell envelope
SAUSA300_2143		3.32	Conserved hypothetical protein
SAUSA300_2178	RpoA	3.23	Transcription
SAUSA300_2058	AtpD	3.13	Energy metabolism
SAUSA300_0527	RpoB	2.87	Transcription
SAUSA300_2059	AtpG	2.80	Energy metabolism
SAUSA300_0528	RpoC	2.63	Transcription
SAUSA300_0194	MurP	2.49	Cellular processes (includes toxins and virulence factors)
SAUSA300_1565		2.40	Central intermediary metabolism
SAUSA300_1685		2.28	Conserved hypothetical protein
SAUSA300_2453	NcaC	2.23	Transport and binding proteins
SAUSA300_2440	FnbB	2.17	Cell envelope
SAUSA300_0724		2.10	Cell envelope
SAUSA300_0514	CysE	2.08	Amino acid biosynthesis
SAUSA300_0963	QoxA	2.05	Energy metabolism
SAUSA300_2573	IsaB	2.00	Unknown function
SAUSA300_1881	GatA	0.50	Protein synthesis
SAUSA300_0691	SaeR	0.49	Regulatory functions
SAUSA300_2469	SdaAA	0.49	Energy metabolism
SAUSA300_0386	Xpt	0.48	Purines, pyrimidines, nucleosides, and nucleotides
SAUSA300_1258		0.48	Energy metabolism
SAUSA300_1293	LysA	0.48	Amino acid biosynthesis
SAUSA300_0325	GcvH	0.48	Energy metabolism
SAUSA300_1360	UbiE	0.48	Protein synthesis
SAUSA300_0480	Pth	0.48	Protein synthesis
SAUSA300_0716		0.48	Purines, pyrimidines, nucleosides, and nucleotides
SAUSA300_2066	Upp	0.47	Purines, pyrimidines, nucleosides, and nucleotides
SAUSA300_0492	FolP	0.47	Biosynthesis of cofactors, prosthetic groups, and carriers
SAUSA300_2076		0.46	Central intermediary metabolism
SAUSA300_1640	Icd	0.46	Energy metabolism
SAUSA300_0841		0.46	Conserved hypothetical protein
SAUSA300_2197	RplP	0.45	Protein synthesis
SAUSA300_1443	RluB	0.45	Protein synthesis
SAUSA300_1159	NusA	0.45	Transcription
SAUSA300_1530	YbeY	0.44	Conserved hypothetical protein
SAUSA300_0820	SufS	0.44	Biosynthesis of cofactors, prosthetic groups, and carriers
SAUSA300_1937		0.44	Mobile and extrachromosomal element functions
SAUSA300_1049	MurI	0.43	Cell envelope
SAUSA300_1679	AcsA	0.43	Central intermediary metabolism
SAUSA300_1178	RecA	0.42	DNA metabolism
SAUSA300_1269	FemA	0.42	Cellular processes (includes toxins and virulence factors)
SAUSA300_1882	GatC	0.41	Signal transduction
SAUSA300_1614	HemL1	0.41	Biosynthesis of cofactors, prosthetic groups, and carriers
SAUSA300_0067		0.41	Unknown function
SAUSA300_1634	CoaE	0.40	Biosynthesis of cofactors, prosthetic groups, and carriers
SAUSA300_1288	DapA	0.40	Amino acid biosynthesis
SAUSA300_1478		0.37	Cell envelope
SAUSA300_1285		0.35	Transport and binding proteins
SAUSA300_0971	PurL	0.35	Purines, pyrimidines, nucleosides, and nucleotides
SAUSA300_0368	RpsR	0.33	Protein synthesis
SAUSA300_1357	AroC	0.33	Amino acid biosynthesis
SAUSA300_0919	MurE	0.32	Cell envelope
SAUSA300_1156	ProS	0.32	Protein synthesis
SAUSA300_0753		0.30	Conserved hypothetical protein
SAUSA300_0741	UvrB	0.29	DNA metabolism
SAUSA300_0692	SaeQ	0.27	Conserved hypothetical protein
SAUSA300_1523		0.27	Conserved hypothetical protein
SAUSA300_2526	PyrD	0.26	Purines, pyrimidines, nucleosides, and nucleotides
SAUSA300_0364	YchF	0.26	Unknown function
SAUSA300_1144	TrmFO	0.24	Unknown function
SAUSA300_1861		0.24	Conserved hypothetical protein
SAUSA300_1007		0.24	Unknown function
SAUSA300_0329		0.24	Unknown function
SAUSA300_0732		0.23	Conserved hypothetical protein
SAUSA300_0538		0.23	Energy metabolism
SAUSA300_2251		0.22	Energy metabolism
SAUSA300_2025	RsbU	0.22	Cellular processes (includes toxins and virulence factors)
SAUSA300_2525		0.21	Conserved hypothetical protein
SAUSA300_2510		0.20	Conserved hypothetical protein
SAUSA300_2312	Mqo	0.18	Energy metabolism
SAUSA300_2296		0.17	Unknown function
SAUSA300_0737	SecA1	0.10	Protein fate
SAUSA300_2477	CidC	0.09	Energy metabolism
SAUSA300_1711	PutA	0.05	Energy metabolism
SAUSA300_2125		0.05	Transport and binding proteins
SAUSA300_0857	PpiB	0.01	Conserved hypothetical protein

^1^ Fold change is based on comparing abundance of proteins in Δ*ppiB*/WT. Fold change >1 is indicative of an increase in abundance in Δ*ppiB* culture supernatants. Fold change <1 is indicative of a decrease in abundance in Δ*ppiB* culture supernatants.

**Table 2:** Proteins with altered abundance in Δ*prsA* culture supernatants.

Gene Designation	Protein Name	Fold Change ^1^	Functional Grouping
SAUSA300_1018		11.13	Conserved hypothetical protein
SAUSA300_0062	ArcB	7.92	Amino acid biosynthesis
SAUSA300_2052		2.96	DNA metabolism
SAUSA300_1606		2.63	Conserved hypothetical protein
SAUSA300_0963	QoxA	2.23	Energy metabolism
SAUSA300_1341	Pbp2	2.06	Cell envelope
SAUSA300_0318	NanE	0.50	Central intermediary metabolism
SAUSA300_1763	EpiP	0.44	Protein fate
SAUSA300_1937		0.44	Mobile and extrachromosomal element functions
SAUSA300_2082	RpoE	0.42	Transcription
SAUSA300_0923	HtrA2	0.38	Protein fate
SAUSA300_0279	EsaA	0.37	Cell envelope
SAUSA300_2032	KdpC	0.32	Transport and binding proteins
SAUSA300_0226	FadB	0.31	Fatty acid and phospholipid metabolism
SAUSA300_1934		0.30	Mobile and extrachromosomal element functions
SAUSA300_1790	PrsA	0.03	Protein fate

^1^ Fold change is based on comparing abundance of proteins in Δ*prsA*/WT. Fold change >1 is indicative of an increase in abundance in Δ*prsA* culture supernatants. Fold change <1 is indicative of a decrease in abundance in Δ*prsA* culture supernatants.

**Table 3:** Proteins interacting with PrsA.

Gene Designation	Protein Name	Fold Change ^1^	Protein Description
SAUSA300_1790	PrsA	15.61748634	Foldase protein PrsA
SAUSA300_1512	Pbp3	9.75	Penicillin-binding protein 3
SAUSA300_0838	DltD	7.25	D-alanine-activating enzyme/D-alanine-D-alanyl, dltD protein
SAUSA300_0958	LcpB	6	Transcriptional regulator
SAUSA300_1588	LytH	6	Probable cell wall amidase LytH
SAUSA300_0963	QoxA	5.636363636	Probable quinol oxidase subunit 2
SAUSA300_1974	LukB	4.833333333	Uncharacterized leukocidin-like protein 1
SAUSA300_0032	MecA	4.5	Penicillin-binding protein 2
SAUSA300_2259	LcpC	4.454545455	Putative transcriptional regulator
SAUSA300_0274		4.285714286	Uncharacterized protein
SAUSA300_0419		3.8125	Uncharacterized lipoprotein
SAUSA300_2136	HtsA	3.705882353	Iron compound ABC transporter, iron compound-binding protein
SAUSA300_1982	GroL	3.702702703	60 kDa chaperonin
SAUSA300_0962	QoxB	3.608695652	Probable quinol oxidase subunit 1
SAUSA300_2578		3.6	Putative phage infection protein
SAUSA300_2213		3.5	AcrB/AcrD/AcrF family protein
SAUSA300_2328		3.333333333	Uncharacterized protein
SAUSA300_2092	Dps	3.142857143	General stress protein 20U
SAUSA300_2100		3.133333333	Lytic regulatory protein
SAUSA300_0992		3.090909091	Putative lipoprotein
SAUSA300_2144		3.083333333	Uncharacterized protein
SAUSA300_0868	SpsB	3	Signal peptidase I
SAUSA300_0279	EsaA	∞	Putative membrane protein
SAUSA300_2579	LytZ	∞	N-acetylmuramoyl-L-alanine amidase domain protein

^1^ Fold change is based on proteins found in association with PrsA in comparison to an empty-vector control. Fold chance >1 indicates greater abundance in PrsA immunoprecipitation samples. Fold change = ∞ indicates protein not detected in negative control.

**Table 4:** Proteins interacting with PpiB.

SAUSA300 Gene Number	Protein Name	Fold Change ^1^	Protein Description
SAUSA300_0857	PpiB	31.5	Putative peptidyl-prolyl cis-trans isomerase
SAUSA300_0737	SecA1	8.5	Protein translocase subunit SecA 1
SAUSA300_1027	RpmF	7.8	50S ribosomal protein L32
SAUSA300_2364	Sbi	7.5	Immunoglobulin-binding protein sbi
SAUSA300_1535	RpsU	6.25	30S ribosomal protein S21
SAUSA300_1178	RecA	5.75	Protein RecA
SAUSA300_0220	PflB	5.5	Formate acetyltransferase
SAUSA300_1193	GlpD	4.88	Aerobic glycerol-3-phosphate dehydrogenase
SAUSA300_1645	PfkA	4.83	ATP-dependent 6-phosphofructokinase
SAUSA300_0757	Pgk	4.83	Phosphoglycerate kinase
SAUSA300_0798		4.63	Lipoprotein
SAUSA300_0388	GuaB	4.5	Inosine-5′-monophosphate dehydrogenase
SAUSA300_1525	GlyQS	4.18	Glycine--tRNA ligase
SAUSA300_0489	FtsH	3.86	ATP-dependent zinc metalloprotease FtsH
SAUSA300_2067	GlyA	3.75	Serine hydroxymethyltransferase
SAUSA300_1880	GatB	3.63	Aspartyl/glutamyl-tRNA (Asn/Gln) amidotransferase subunit B
SAUSA300_0491	CysK	3.63	Cysteine synthase
SAUSA300_1150	Tsf	3.62	Elongation factor Ts
SAUSA300_0389	GuaA	3.56	GMP synthase [glutamine-hydrolyzing]
SAUSA300_1684		3.25	Uncharacterized protein
SAUSA300_0533	Tuf	3.09	Elongation factor Tu
SAUSA300_0693	SaeP	3.07	Putative lipoprotein
SAUSA300_1459	Gnd	3.06	6-phosphogluconate dehydrogenase, decarboxylating
SAUSA300_0496	LysS	3	Lysine--tRNA ligase
SAUSA300_0716	NrdE	∞	Ribonucleoside-diphosphate reductase
SAUSA300_2251		∞	Dehydrogenase family protein
SAUSA300_1881	GatA	∞	Glutamyl-tRNA (Gln) amidotransferase subunit A
SAUSA300_1586	AspS	∞	Aspartate--tRNA ligase
SAUSA300_1167	Pnp	∞	Polyribonucleotide nucleotidyltransferase
SAUSA300_0009	SerS	∞	Serine--tRNA ligase
SAUSA300_1629	ThrS	∞	Threonine--tRNA ligase
SAUSA300_2214	FemX	∞	Lipid II:glycine glycyltransferase

^1^ Fold change is based on proteins found in association with PpiB in comparison to an empty-vector control. Fold chance >1 indicates greater abundance in PpiB immunoprecipitation samples. Fold change = ∞ indicates protein not detected in negative control.

**Table 5:** Strains and plasmids used in this study.

Name	Characteristics	Source
Strains		
*S. aureus*		
RN4220	Restriction-deficient transformation recipient	[43]
TCH1516	Community-associated USA300 MRSA isolate	[44]
RKC0323	TCH1516 Δ*ppiB*	[21]
RKC0085	TCH1516 Δ*prsA*	[24]
RKC0183	TCH1516 *hla*::Bursa, *hla* mutant	(21)
RKC0374	TCH1516 Δ*ppiB* pMK4_*ppiB*-HA	This work
RKC0536	TCH1516 Δ*ppiB* pMK4	This work
RKC0283	TCH1516 Δ*prsA* pMK4_*prsA*-his	(24)
RKC0129	TCH1516 Δ*prsA* pMK4	(24)
JE2	USA300 LAC isolate cured of plasmids LAC-p01 and LAC-p03	[45]
NE1354	USA300 JE2 *hla*::Bursa, *hla* NTML transposon mutant	[45]
AH1263	USA300 Lac isolate cured of plasmid Lac-p03	[46]
RKC0521	AH1263 *hla*::Bursa, *hla* mutant	[47]
RKC0753	AH1263 Δ*αPSMs*	This work
Plasmids		
pMK4	Gram-positive shuttle vector (Cm^r^)	[48]
pRKC0131	pMK4_*ppiB*-HA (vector overexpressing *ppiB* with an HA tag)	[24]
pRKC0126	pMK4_prsA-His (vector overexpressing prsA with a poly-histidine tag)	[24]
pRKC0674	pJB38 containing DNA flanking αPSM transcript with ery cassette	This work

**Table 6:** Oligonucleotides used in this study.

Name	Sequence
#0273	GGTGCTGGGCAAATACAAGT (*gyrB*)
#0274	TCCCACACTAAATGGTGCAA (*gyrB*)
#0263	TGCAAATGTTTCGATTGGTC (*hla*)
#0264	CCCCAATTTTGATTCACCATA (*hla*)
#0271	ACAGGAGGACAAAACGATGG (*psmα*)
#0272	CCCTATTGGTATAGTGGCCTGA (*psmα*)
#0490	CAAGACGTCCGTCGGTCTACCTTTCCATGC
#0493	GGGGTACCACGTGGCACTTTCCAAAAAC
#0638	CCGGAATTCGCTCCTTGGAAGCTGTCAGT
#0639	AAAACTGCAGGAAGCAAACTTAAGAGTGTGTTGA
#0644	CCGGAATTCGATGTGAGGTGAGTCTTGTTAGTTTG
#0645	AAAACTGCAGAGATTACCTCCTTTGCTTATGAGT

## Supplementary Materials

The following are available online at https://www.mdpi.com/2072-6651/11/6/343/s1, Figure S1: The αPSMs are the primary toxins responsible for human erythrocyte lysis while Hla is the primary toxin active against rabbit erythrocytes. Figure S2: RT-qPCR analysis of *hla* and *α*PSM transcript levels in, W.T.; *ΔppiB,* and *ΔprsA* strains. Table S1: PpiB secreted proteome analysis. Table S2: PrsA secreted proteome analysis. Table S3: PrsA immunoprecipitation. Table S4: PpiB immunoprecipitation.
