# Supplementary material for: Novel Regulation of Alpha-Toxin and the Phenol-Soluble Modulins by Peptidyl-Prolyl cis/trans Isomerase Enzymes in Staphylococcus aureus
Source: Toxins (Basel). 2019 Jun 16;11(6):343. doi: 10.3390/toxins11060343 (PMC6628628; doi:10.3390/toxins11060343)
Supplement: Supplementary file 1 [file toxins-11-00343-s001.zip › toxins-506015-supple-final.docx]

Supplementary Materials: Novel Regulation of Alpha-Toxin and the Phenol-Soluble Modulins by Peptidyl-Prolyl cis/trans Isomerase Enzymes in Staphylococcus aureus

Rebecca A. Keogh, Rachel L. Zapf, Emily Trzeciak, Gillian G. Null, Richard E Wiemels and
Ronan K. Carroll


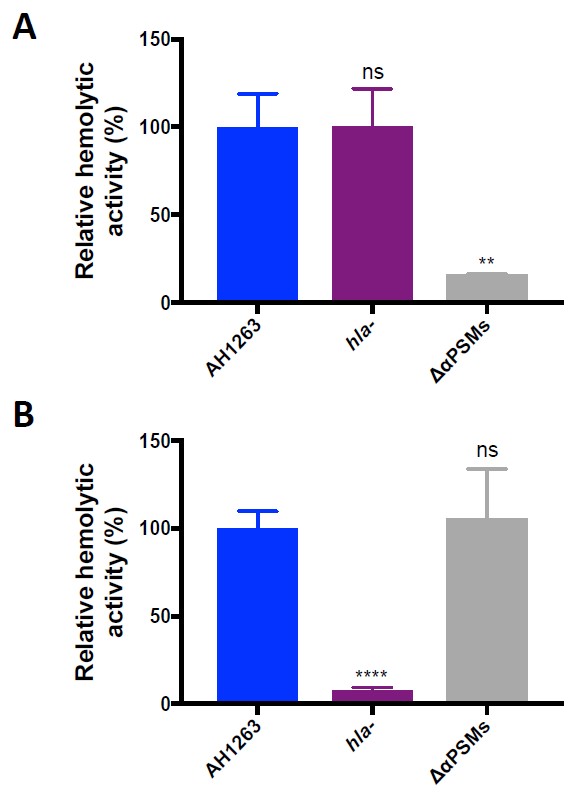


**Figure 1.** The αPSMs are the primary toxins responsible for human erythrocyte lysis while. Hla is the primary toxin ac < ve against rabbit erythrocytes. (**A**) A decrease in hemoly < c ac < vity against human erythrocytes was observed using culture supernatants from an *αPSM* mutant strain. (**B**) A decrease in hemoly < c ac < vity against rabbit erythrocytes was observed using culture supernatants from an *hla* mutant.Significance was determined by Student's *t* test. **** *p* < 0.001; ** *p* < 0.01; ns, not significant.


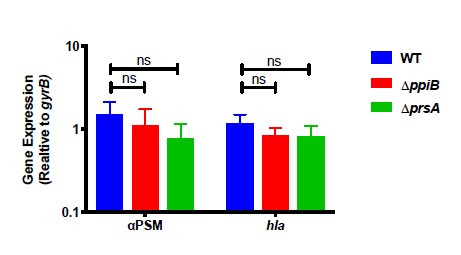


Figure S2: RT-qPCR analysis of hla and αPSM transcript levels in, W.T.; ΔppiB, and ΔprsA strains.
